# Exploring and Predicting HIV Preexposure Prophylaxis Adherence Patterns Among Men Who Have Sex With Men: Randomized Controlled Longitudinal Study of an mHealth Intervention in Western China

**DOI:** 10.2196/58920

**Published:** 2024-12-12

**Authors:** Bing Lin, Jiayan Li, Jiaxiu Liu, Wei He, Haiying Pan, Xiaoni Zhong

**Affiliations:** 1 School of Public Health, Chongqing Medical University, Chongqing, China; 2 Research Center for Medicine and Social Development, Chongqing, China; 3 School of Medical Informatics, Chongqing Medical University, Chongqing, China; 4 Jiulongpo District Center for Disease Control and Prevention, Chongqing, China

**Keywords:** preexposure prophylaxis, adherence, trajectory analysis, men who have sex with men, mHealth, mobile health, mHealth intervention, decision tree

## Abstract

**Background:**

Preexposure prophylaxis (PrEP) is an effective strategy to reduce the risk of HIV infection. However, the efficacy of PrEP is highly dependent on adherence. Meanwhile, adherence changes over time, making it difficult to manage effectively.

**Objective:**

Our study aimed to explore and predict the patterns of change in PrEP adherence among men who have sex with men (MSM) and evaluate the impact of the WeChat-based reminder intervention on adherence, thus providing more information for PrEP implementation strategies.

**Methods:**

From November 2019 to June 2023, in a randomized controlled longitudinal study of the PrEP demonstration project in Western China (Chongqing, Sichuan, and Xinjiang) based on a mobile health (mHealth) reminder intervention, participants were randomly divided into reminder and no-reminder groups, with those in the reminder group receiving daily reminders based on the WeChat app. Participants were followed up and self-reported their medication adherence every 12 weeks for a total of 5 follow-up visits. We used the growth mixture model (GMM) to explore potential categories and longitudinal trajectories of adherence among MSM, and patterns of change in PrEP adherence were predicted and evaluated based on the decision tree.

**Results:**

A total of 446 MSM were included in the analysis. The GMM identified 3 trajectories of adherence: intermediate adherence group (n=34, 7.62%), low adherence ascending group (n=126, 28.25%), and high adherence decline group (n=286, 64.13%). We included 8 variables that were significant in the univariate analysis in the decision tree prediction model. We found 4 factors and 8 prediction rules, and the results showed that HIV knowledge score, education attainment, mHealth intervention, and HIV testing were key nodes in the patterns of change in adherence. After 10-fold cross-validation, the final prediction model had an accuracy of 75%, and the classification accuracy of low and intermediate adherence was 78.12%.

**Conclusions:**

The WeChat-based reminder intervention was beneficial for adherence. A short set of questions and prediction rules, which can be applied in future large-scale validation studies, aimed at developing and validating a short adherence assessment tool and implementing it in PrEP practices among MSM.

## Introduction

In China, men who have sex with men (MSM) are the high-risk population for HIV infection. Previous studies have estimated the overall national HIV prevalence among MSM in China to be 5.7% from 2001 to 2018 [1]. The annual number of newly diagnosed HIV infections through homosexual transmission in China increased from 2.5% in 2006 to 25.6% in 2022 [2]. To reduce the persistently high incidence of new infections among high-risk populations, we need to focus on HIV prevention. Preexposure prophylaxis (PrEP) is a biomedical HIV prevention intervention that consists primarily of the prophylactic daily use of antiretroviral medications to reduce the risk of infection in the event of HIV exposure [3]. Effective PrEP services for at-risk populations, including MSM, are key to reducing new HIV infections [4].

However, a growing number of studies have shown that the efficacy of PrEP is highly dependent on medication adherence [5,6]. PrEP adherence is defined as the users taking the medication as prescribed by the clinician, occurring behaviors consistent with the clinician’s orders, and vice versa, known as nonadherence. A previous systematic review and meta-analysis study showed that the PrEP strategy was highly effective among MSM. Meanwhile, PrEP efficacy was strongly correlated with adherence, with the RR decreasing by 0.13 as adherence increased from 50% to 60%. It was shown that, on average, a 10% decrease in adherence would correspond to a 13% decrease in PrEP efficacy [7]. In summary, achieving a high level of adherence is necessary for PrEP to be completely effective. This highlights the need for good adherence in ensuring the efficacy of PrEP among the MSM population.

Moreover, earlier studies have acknowledged the time-varying nature of adherence, both within and between individuals over time [8]. Exploring and elucidating patterns of change in adherence can contribute to understanding the reasons for users’ behavioral changes and enhance the capacity to recognize individuals who are more susceptible to nonadherence. Studies assessing long-term behavioral patterns of PrEP adherence have been conducted in many countries, including Australia [9], America [10], and South Africa [11]. However, at present, few studies in China have assessed patterns of change in adherence. Taken together, several previous empirical studies have shown that adherence is critical for PrEP efficacy in high-risk populations and deserves further attention. Future PrEP strategies will be more effective if we are able to accurately recognize patterns of change in adherence and develop predictive models that reliably identify individuals who engage in nonadherent behavior. Therefore, our study aimed to explore and predict the patterns of change in PrEP adherence based on the decision tree model, thus providing more information for PrEP implementation strategies. Our study was a preliminary application of decision tree prediction modeling to PrEP adherence among the MSM population in Western China. More importantly, recent simulation studies have shown that machine learning techniques, for example, decision tree modeling, can be reliably applied to relatively small samples and are more applicable to our particular study population [12,13].

In addition, mobile health (mHealth) has emerged as a promising tool for improving health care access and delivery globally [14]. Previous studies have shown that mHealth could reach and engage MSM in HIV prevention and care, which suggested that mHealth interventions have the potential to improve HIV prevention efforts [15]. At the same time, willingness to use mHealth intervention for HIV prevention was high among the MSM population [16]. Although research on adherence interventions for PrEP was in its early stages, some mHealth intervention methods showed promise. The WeChat app (Tencent Holdings Ltd) is the most popular social media platform in China, with 1.27 billion monthly active users [17]. Thus, a WeChat-based medication reminder service is a low-cost and scalable intervention.

In summary, based on the randomized controlled longitudinal study of the PrEP demonstration project through the mHealth intervention, the aims of our study were to (1) identify patterns of change in PrEP adherence among the MSM population; (2) provide an initial basis for identifying a set of questions and decision rules that can accurately predict individuals at high risk for low PrEP adherence behaviors among the MSM population based on the decision tree prediction model; and (3) evaluate the impact of the mHealth intervention on adherence. Validation in larger samples in the future could contribute to the creation of a comprehensive, brief adherence screening tool based on the results of our study. In the long term, our study will help administrators target individuals with low adherence to preventive interventions and, at the same time, provide a direct basis for policy development and service planning for the implementation of PrEP among MSM in China, which is of great practical significance.

## Methods

### Participants and Procedure

From November 2019 to June 2023, our study was conducted among the MSM population in Western China (Chongqing, Sichuan, and Xinjiang). The randomized controlled longitudinal study of the PrEP demonstration project was based on a mobile phone intervention, accompanied by the Chinese 13th Five-Year Plan for AIDS Prevention and Control carried out by the Ministry of Science and Technology (Chinese Clinical Trial Registration Number: ChiCTR1900026414).

The participants were HIV-negative MSM individuals at higher risk of HIV infection. We recruited eligible MSM individuals through collaboration with local nongovernmental organizations and peer referrals. The inclusion criteria included (1) assigned male sex at birth; (2) age 18‐65 years; (3) negative HIV antigen antibody test; (4) self-reported high-risk sexual activity with a male partner (both temporary and regular) in the last 6 months; (5) no serious heart, liver, or kidney disease or hematopoietic dysfunction, bleeding tendency, and bleeding disease; (6) willing to use the experimental drug under guidance and comply with follow-up arrangements; and (7) signed informed consent.

After completing the baseline questionnaire, participants who met the inclusion criteria were randomly divided into a reminder group and a no-reminder group. Participants in the reminder group received daily mobile phone–based reminder messages and took lamivudine-tenofovir (PrEP drug) orally daily. The reminder information relies on the internet and an intelligent medication information tracking management system using cloud computing, big data, intelligent hardware, and other new-generation IT products to collect medication plans, medication reminders, medication logs, electronic instructions for medicines, adverse reactions, health knowledge, and other process information, and through the collection of information, it provides information for the administrator to provide the user with effective medication management to achieve the expected preventive effect. The reminder messages were sent at regular times daily through the WeChat app to remind users to take PrEP. At the same time, in order to protect the user’s privacy, the content of the messages we sent were “processed.” For example, when users received “You need to learn,” it meant that they were reminded to take medication; “Time for examination” meant that it was time for the next follow-up visit. The presentation of the reminder messages on WeChat is shown in Figures S1 and S2 in Multimedia Appendix 1. Participants in the no-reminder group who took PrEP daily did not receive reminder messages. Participants were followed up and self-reported their medication adherence every 12 weeks for a total of 5 follow-up visits.

### Measurement

Demographic variables of MSM included age, place of residence, ethnicity, education attainment, employment status, marital status, and monthly disposable income.

The HIV Knowledge Scale (Cronbach *α*=.672) was used to measure knowledge mastery in the MSM population. The scale consisted of 13 questions and was based on the revised Universal Scale of the International HIV Knowledge Survey [18]. The answers included “correct,” “wrong,” and “I don’t know.” One point was awarded for a correct answer and zero points for an incorrect or unknown answer. A higher score indicated a higher level of knowledge about HIV. We believed that a score ≥11 indicates a high level of knowledge [19,20].

Meanwhile, we also measured the level of risk perception of the participants. They were asked, “How likely do you think you are to contract HIV? How serious do you think AIDS is? How much of a threat do you think AIDS poses to you?” These 3 items were used to measure their perceived risk. Participants rated their perceived risk on a scale of 1‐5, with 1‐2 representing a low level of perception, 3 representing a moderate level of perception, and 4‐5 representing a high level of perception.

Previous literature mentioned that MSM may show different preferences and willingness to use PrEP and other biomedical prevention strategies based on the role of anal sex [21]. Therefore, our study included the variable “sexual role” to explore whether differences in sexual roles might lead to differences in adherence to PrEP in the MSM population. Sexual role was the way of performing sexual behavior with a male sexual partner, such as inserter (like “top”) and receiver (like “bottom”).

We also collected information about HIV testing, HIV counseling, the number of sexual partners, condom use, internet searches for sexual partners, sexually transmitted diseases history, commercial sex, recreational drug use, attitudes, and potential effects of PrEP use among male sexual partners among the participants.

### Adherence

During the follow-up, participants self-reported the number of days they missed taking their medication in the previous 2 weeks, and adherence was equal to the proportion of days medication was taken.

### Growth Mixture Model

The growth mixture model (GMM) was used in our study to determine potential class trajectories for adherence. The model used Akaike information criteria (AIC), Bayesian information criteria (BIC), sample size–adjusted BIC (aBIC), and entropy to explore the optimal trajectory of internalization and externalization problems. The AIC, BIC, and aBIC statistics were all used to determine how well the model fits by comparing the difference between the expected and actual values, with lower values indicating a good model fit. The entropy took values in the range 0‐1, with closer to 1 indicating a clearer group classification. The Vuong-Lo-Mendell-Rubin likelihood ratio test and bootstrapped likelihood ratio test were used to compare the differences in fit between the k-1 and k-category models. When the *P* value was significant, the model was considered well fitted for the k-category.

### Decision Tree

The decision tree is based on the known probability of the occurrence of various situations, through the composition of the decision rule to determine the probability and evaluate and judge the risk decision analysis method, which is the intuitive use of probability analysis using a graphical method. This decision branching is drawn graphically, much like the branches of a tree. The top decision node in the tree is called the root node; it corresponds to the best predictor. The final nodes are called terminal nodes or leaves. The numbers on the terminal nodes indicate the probability of each class and are what determine the final classification. In our study, the Gini index was used as a criterion for selecting features. The magnitude of the Gini index indicated the complexity of the data, and when the Gini index was less than a threshold, it indicated that the purity of the data was greater and the nodes of the tree stopped splitting. The decision tree model was pruned according to the complexity parameter (Cp) value, and the Cp value corresponding to the smallest error was selected to obtain the optimal decision tree model.

### Statistical Analysis

Longitudinal data collected on repeated measures of PrEP adherence among the MSM population were analyzed using the GMM to explore potential categories and longitudinal trajectories of adherence, and statistical metrics were used to measure the degree of model fit. Based on the different potential categories of adherence, relevant information was compared using univariate analysis, and predictors with a *P* value ≤.05 were screened and included in the decision tree prediction model. The SMOTE algorithm in the “smotefamily” package of the R program was used to balance the adherence classification data; the “rpart” package was used to construct the decision tree prediction model. The predictive power of the final model was assessed by three criteria: (1) “accuracy” indicates the overall percentage of correct classifications; (2) “positive classification accuracy” refers to the percentage of predictions that the model accurately classifies as positive; and (3) “negative classification accuracy” refers to the percentage of predictions that the model accurately classifies as negative. For each of the 3 evaluation criteria, 10-fold cross-validation was used, and their estimates were computed as the average of the 10 test samples. At the same time, missing values were considered to be informative indicators, and therefore were considered as potential predictors in modeling [22]. *P* value ≤.05 represented a statistical difference. All analyses were completed using Mplus (Muthén & Muthén) and R software (R Foundation for Statistical Computing).

### Ethical Considerations

The study was approved by the Ethics Committee of Chongqing Medical University (reference number: 2019001). Before participating in this research, the participants were fully informed about the purpose, significance, voluntary participation, and confidentiality of the research. Each participant signed an informed consent form.

## Results

### Basic Demographic Information

**Figure 1.**
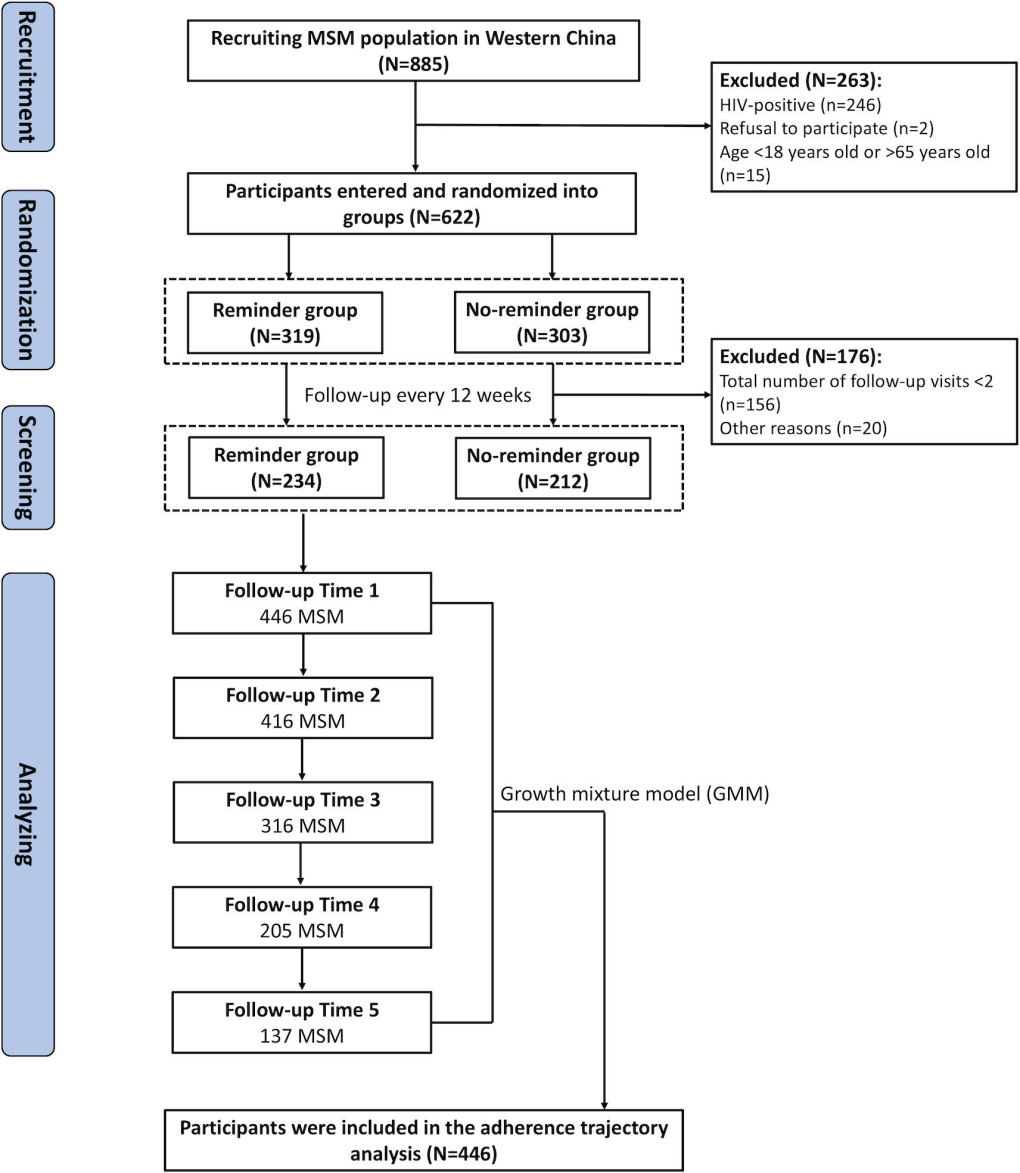
Flowchart for the screening of study participants. MSM: men who have sex with men; GMM: growth mixture model.

A total of 885 MSM were recruited in Western China. Of these, 263 MSM were excluded because they did not meet the requirements for inclusion in the cohort. A total of 622 MSM were enrolled in the longitudinal study and were randomized into a reminder group (n=319) and a no-reminder group (n=303). Due to the need to model longitudinal trajectories of adherence, we excluded a subset of participants with less than 2 follow-up visits. A total of 446 MSM were eventually included in the statistical analysis, with 234 and 212 MSM in the reminder and no-reminder groups, respectively. The detailed screening process is shown in Figure 1. We compared basic demographic information between the reminder and no-reminder groups (Table S1 in Multimedia Appendix 1). According to the results of the univariate analysis, none of the variables differed statistically in both groups. We performed descriptive analyses of the number of MSM and adherence in each follow-up period (Table S2 in Multimedia Appendix 1).

### Exploring Patterns of Change in Adherence

**Figure 2.**
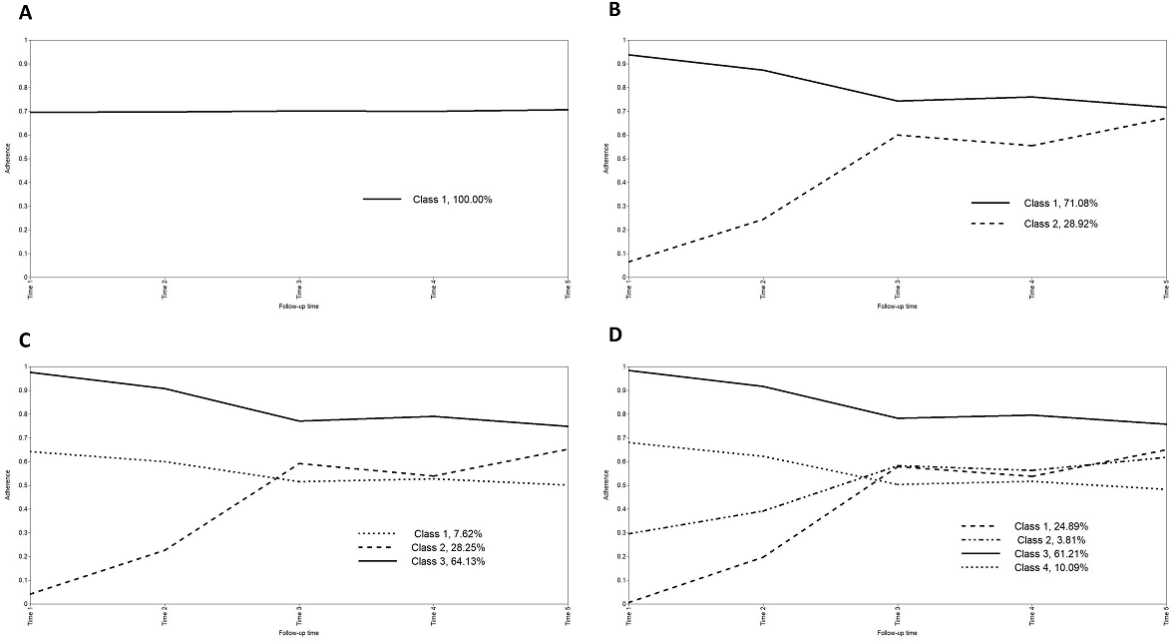
Trajectory of PrEP adherence potential categories in the MSM population. A, B, C, and D correspond to schemes with 1, 2, 3, and 4 adherence potential categories, respectively. PrEP: preexposure prophylaxis; MSM: men who have sex with men.

Based on the GMM, we explored 1, 2, 3, and 4 potential categories of adherence (Table 1). Considering the parameters, the scheme for category 4 is reasonable. However, Class 2 accounted for 3.81% (n=17) of the scheme for category 4, which is too small a number to carry out subsequent analyses, and the trajectories of Class 1 and Class 2 were partially close to overlapping. Therefore, we chose the scheme with class probability and trajectory distribution, which was more reasonable for category 3. The parameters of the model also showed good model fit. The adherence trajectories for each categorical scheme were plotted in Figure 2.

**Table 1.** Exploration of potential categories for PrEP^a^ adherence among MSM^b^ population based on GMM^c^.

Class	AIC^d^	BIC^e^	aBIC^f^	Entropy	VLRT^g^	BLRT^h^	Class probability
1C^i^	1119.749	1168.953	1130.870	—^j^	—	—	1
2C	550.175	611.679	564.076	0.989	<.001	<.001	0.7108/0.2892
3C	356.855	430.661	373.537	0.972	<.001	<.001	0.0762/0.2825/0.6413
4C	145.987	232.094	165.449	0.969	<.001	<.001	0.2489/0.0381/0.6121/0.1009

a PrEP: preexposure prophylaxis.

b MSM: men who have sex with men.

c GMM: growth mixture model.

d AIC: Akaike information criteria.

e BIC: Bayesian information criteria.

f aBIC: sample size–adjusted BIC.

g VLRT: Vuong-Lo-Mendell-Rubin likelihood ratio test.

h BLRT: bootstrapped likelihood ratio test.

i C: category.

j Not applicable.

Based on the descriptions of the 3 latent categories of adherence (Table 2), Class 1 (n=34, 7.62%) had mean values of intercept (I) and slope (S) of 0.642 (*P*<.001) and –0.042 (*P*=.35), respectively, and was named “Intermediate adherence group.” Class 2 (n=126, 28.25%) had mean values of intercept (I) and slope (S) of 0.042 (*P*<.001) and 0.183 (*P*<.001), respectively, and was named “Low adherence ascending group.” Class 3 (n=286, 64.13%) had mean values of intercept (I) and slope (S) of 0.976 (*P*<.001) and –0.068 (*P*<.001), respectively, and was named “High adherence decline group.”

**Table 2.** Description of GMM^a^ parameters for 3 potential categories of PrEP^b^ adherence among MSM^c^ population.

Variables			Estimate	SE	*P* value
Class 1: Intermediate adherence group					
Mean I^d^			0.642	0.039	<.001
Mean S^e^			–0.042	0.045	.35
Class 2: Low adherence ascending group					
Mean I			0.042	0.010	<.001
Mean S			0.183	0.020	<.001
Class 3: High adherence decline group					
Mean I			0.976	0.007	<.001
Mean S			–0.068	0.011	<.001

a GMM: growth mixture model.

b PrEP: preexposure prophylaxis.

c MSM: men who have sex with men.

d I: intercept.

e S: slope.

### Differences Between Adherence Potential Categories

We performed a univariate analysis of the relevant variables (Table 3). According to the results of the *χ*^2^ test, educational attainment (*P*=.007), HIV knowledge score (*P*<.001), HIV testing (*P*=.05), HIV counseling (*P*=.02), number of male sexual partners (*P*=.003), attitudes of male sexual partners (*P*=.04), PrEP use (*P*=.05), and groups (reminder and no-reminder groups, *P*=.01) showed significant variability between patterns of change in adherence.

**Table 3.** Univariate analysis of different latent categories of PrEP^a^ adherence among MSM^b^ population.

Variables	Total (N=446)	Intermediate adherence group (n=34)	Low adherence ascending group (n=126)	High adherence decline group (n=286)	*P* value
Age, n (%)					.18
18‐25	70 (15.70)	8 (23.53)	26 (20.63)	36 (12.59)	
25‐35	179 (40.13)	11 (32.35)	48 (38.10)	120 (41.96)	
≥35	197 (44.17)	15 (44.12)	52 (41.27)	130 (45.45)	
Place of residence, n (%)^c^					.39
Urban	329 (74.43)	27 (79.41)	87 (70.16)	215 (75.70)	
Rural	113 (25.57)	7 (20.59)	37 (29.84)	69 (24.30)	
Ethnicity, n (%)					.39
Ethnic Han	413 (92.60)	33 (97.06)	114 (90.48)	266 (93.01)	
Ethnic minorities	33 (7.40)	1 (2.94)	12 (9.52)	20 (6.99)	
Education attainment, n (%)					.007^d^
Primary school or below	4 (0.90)	1 (2.94)	3 (2.38)	0 (0.00)	
Junior high	24 (5.38)	0 (0.00)	11 (8.73)	13 (4.55)	
High school or vocational high school	93 (20.85)	8 (23.53)	32 (25.40)	53 (18.53)	
College or university degree or above	325 (72.87)	25 (73.53)	80 (63.49)	220 (76.92)	
Employment status, n (%)					.82^d^
Employed	384 (86.10)	28 (82.36)	108 (85.71)	248 (86.72)	
Unemployed or retired	33 (7.40)	4 (11.76)	10 (7.94)	19 (6.64)	
Student	29 (6.50)	2 (5.88)	8 (6.35)	19 (6.64)	
Marital status, n (%)					.53
Married	60 (13.45)	3 (8.82)	15 (11.90)	42 (14.69)	
Unmarried or divorced	386 (86.55)	31 (91.18)	111 (88.10)	244 (85.31)	
Monthly disposable income, n (%)^c^					.69
1000‐3000 CNY^e^	104 (23.37)	7 (20.59)	35 (28.00)	62 (21.68)	
3000‐10,000 CNY	310 (69.66)	25 (73.53)	82 (65.60)	203 (70.98)	
≥10,000 CNY	31 (6.97)	2 (5.88)	8 (6.40)	21 (7.34)	
HIV knowledge score, n (%)					<.001
<11	204 (45.74)	12 (35.29)	80 (63.49)	112 (39.16)	
≥11	242 (54.26)	22 (64.71)	46 (36.51)	174 (60.84)	
HIV testing, n (%)^c^					.05
Yes	360 (82.38)	30 (88.24)	88 (75.21)	242 (84.62)	
No	77 (17.62)	4 (11.76)	29 (24.79)	44 (15.38)	
HIV counseling, n (%)					.02
Yes	327 (73.32)	24 (70.59)	81 (64.29)	222 (77.62)	
No	119 (26.68)	10 (29.41)	45 (35.71)	64 (22.38)	
How likely do you think you are to contract HIV?, n (%)					.78
Low level	263 (58.97)	21 (61.76)	79 (62.70)	163 (56.99)	
Moderate level	140 (31.39)	9 (26.48)	37 (29.37)	94 (32.87)	
High level	43 (9.64)	4 (11.76)	10 (7.93)	29 (10.14)	
How serious do you think AIDS is?, n (%)^c^					.54^d^
Low level	1 (0.23)	0 (0.00)	1 (0.83)	0 (0.00)	
Moderate level	42 (9.68)	3 (9.68)	10 (8.26)	29 (10.28)	
High level	391 (90.09)	28 (90.32)	110 (90.91)	253 (89.72)	
How much of a threat do you think AIDS poses to you?, n (%)^c^					.61^d^
Low level	40 (9.01)	4 (11.76)	13 (10.40)	23 (8.07)	
Moderate level	61 (13.74)	3 (8.82)	14 (11.20)	44 (15.44)	
High level	343 (77.25)	27 (79.42)	98 (78.40)	218 (76.49)	
Sexual role, n (%)^c^					.97
Mainly “bottom”	124 (28.51)	8 (25.00)	32 (27.12)	84 (29.47)	
Both	88 (20.23)	7 (21.88)	25 (21.19)	56 (19.65)	
Mainly “top”	223 (51.26)	17 (53.12)	61 (51.69)	145 (50.88)	
Number of male sexual partners last month (both temporary and regular), n (%)^c^					.003
0	41 (9.31)	3 (8.83)	22 (17.89)	16 (5.65)	
1	251 (57.05)	21 (61.76)	63 (51.22)	167 (59.01)	
2 or more	148 (33.64)	10 (29.41)	38 (30.89)	100 (35.34)	
Condom use during sex with a male partner, n (%)^c^					.58
Use every time	298 (70.78)	24 (77.42)	74 (65.49)	200 (72.20)	
Sometime use	92 (21.86)	5 (16.13)	28 (24.78)	59 (21.30)	
Never use	31 (7.36)	2 (6.45)	11 (9.73)	18 (6.50)	
Number of female sexual partners last month (both temporary and regular), n (%)^c^					.51^d^
0	368 (85.98)	26 (83.87)	96 (82.76)	246 (87.54)	
1	41 (9.58)	4 (12.90)	12 (10.34)	25 (8.90)	
2 or more	19 (4.44)	1 (3.23)	8 (6.90)	10 (3.56)	
Internet searches for sexual partners, n (%)^c^					.67
No	152 (36.19)	10 (30.30)	47 (38.52)	95 (35.85)	
Yes	268 (63.81)	23 (69.70)	75 (61.48)	170 (64.15)	
Have been diagnosed by a doctor with an STD^f^ (eg, syphilis, genital herpes, gonorrhea, etc), n (%)^c^					.18
Yes	31 (7.03)	5 (14.71)	7 (5.74)	19 (6.67)	
No	410 (92.97)	29 (85.29)	115 (94.26)	266 (93.33)	
Commercial sex, n (%)^c^					.91^d^
Yes	15 (3.39)	1 (2.94)	5 (4.00)	9 (3.17)	
No	428 (96.61)	33 (97.06)	120 (96.00)	275 (96.83)	
Recreational drug use, n (%)^c^					.22^d^
No	435 (97.97)	34 (100.00)	119 (95.97)	282 (98.60)	
Yes	9 (2.03)	0 (0.00)	5 (4.03)	4 (1.40)	
What would be the attitude of the male sexual partner if they knew you were using PrEP?, n (%)					.04
Negative	43 (9.64)	3 (8.82)	18 (14.29)	22 (7.69)	
Neutral	181 (40.58)	16 (47.06)	58 (46.03)	107 (37.41)	
Positive	222 (49.78)	15 (44.12)	50 (39.68)	157 (54.90)	
Do male sexual partner attitudes potentially affect your use of PrEP?, n (%)^c^					.05
No	216 (49.32)	15 (44.12)	50 (42.37)	151 (52.80)	
Neutral	89 (20.32)	5 (14.71)	34 (28.81)	50 (17.48)	
Yes	133 (30.37)	14 (41.18)	34 (28.81)	85 (29.72)	
Group, n (%)					.01
Reminder group	234 (52.47)	19 (55.88)	52 (41.27)	163 (59.99)	
No-reminder group	212 (47.53)	15 (44.12)	74 (58.73)	123 (43.01)	

a PrEP: preexposure prophylaxis

b MSM: men who have sex with men.

c Indicates missing data.

d Indicates the Fisher exact test.

e CNY: Chinese yuan (¥1=US $0.14).

f STD: sexually transmitted disease.

### Predicting the Patterns of Change in Adherence

**Figure 3.**
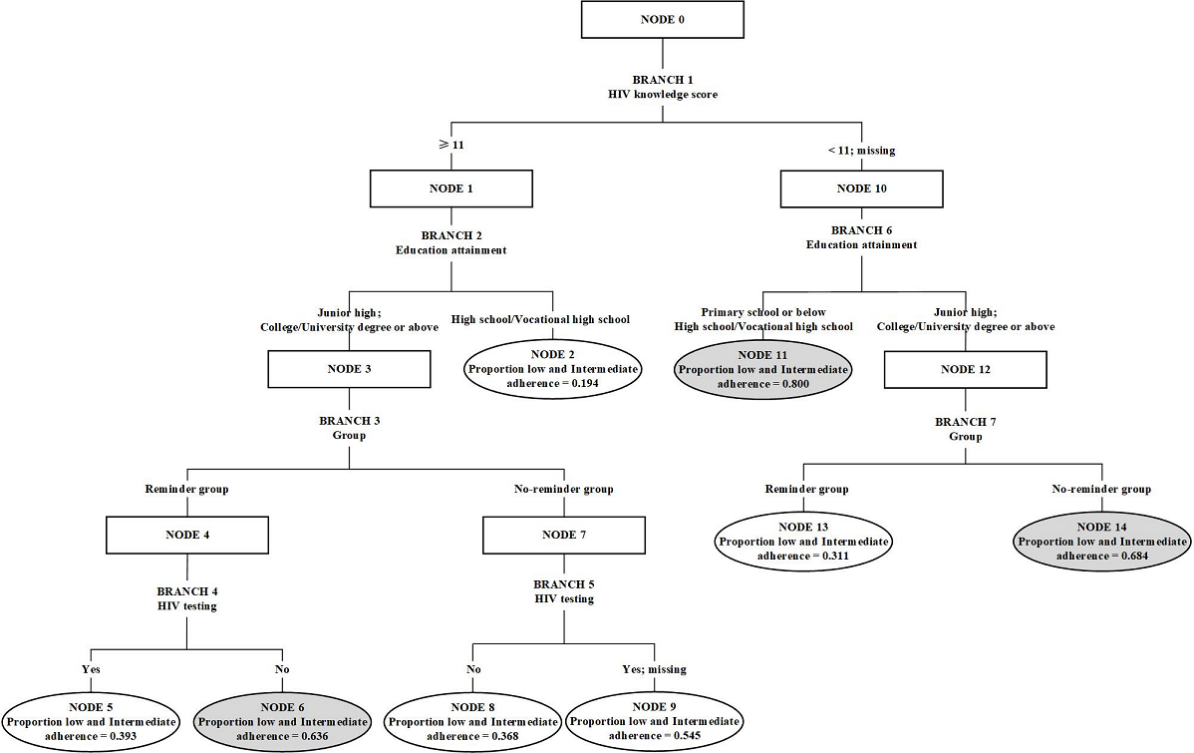
Analytical results of the decision tree prediction model.

In general, only levels of adherence ≥80% are recognized as having a preventive effect [23,24]. For the “intermediate adherence group” and the “low adherence ascending group,” both groups had adherence rates less than 80% and were considered to be at high risk for inadequate prevention. In our modeling, we prioritized these 2 groups together for prediction. We included all 8 variables that were significant in the univariate analysis in the data-balanced decision tree prediction model. The results of the decision tree analysis are shown in Figure 3.

**Figure 4.**
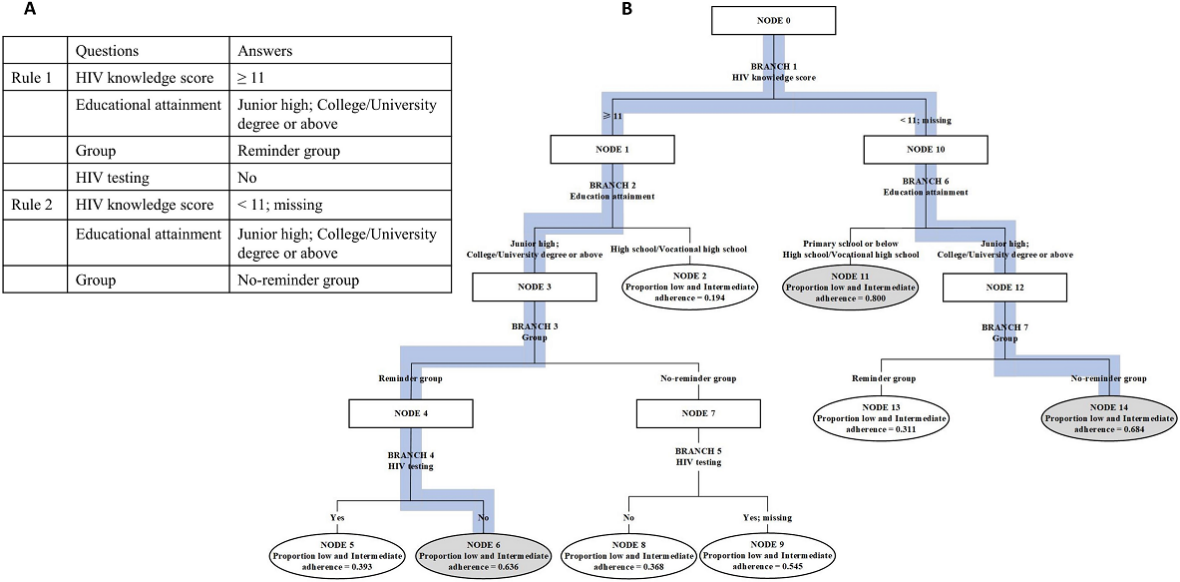
Two prediction rules of the decision tree prediction model. “A” indicates the description of the prediction path, and “B” indicates the prediction path in the decision tree.

In the final model, HIV knowledge score, education attainment, reminder interventions, and HIV testing were key nodes in the patterns of change in adherence. We extracted a total of 4 questions and 8 prediction rules. For example, we selected 2 of these rules for elaboration (Figure 4). If the MSM’s HIV knowledge score (NODE 0) was ≥11, education level (NODE 1) was “Junior high; College or University degree or above,” the MSM belonged to the reminder group (NODE 3), and the MSM had not been tested for HIV (NODE 4), the MSM had a 0.636 probability of belonging to the “low and intermediate adherence” groups. For the second prediction rule, if the MSM’s HIV knowledge score (NODE 0) was <11, education level (NODE 10) was “Junior high; College or University degree or above,” and the MSM did not receive mobile phone–based daily reminders (NODE 12), there was a 0.684 probability that the MSM would belong to the “low and intermediate adherence” groups.

After 10-fold cross-validation, the final prediction model had an accuracy of 75%; the classification accuracy of “low and intermediate adherence” was 78.12%, and that of “high adherence decline group” was 71.43%.

## Discussion

### Principal Findings

Our study was one of the few in China to assess and predict patterns of change in PrEP adherence among the MSM population. The results of the decision tree prediction model showed good and stable prediction results, identifying individuals at risk for low levels of adherence. At the same time, our research results also indirectly demonstrated the role of mHealth in PrEP adherence interventions. It revealed the great potential of digital health tools (for example, mobile phone apps or web-based) and interventions for HIV prevention and overall well-being in the MSM community.

### Three Adherence Potential Categories Among MSM

We selected a 3-category scheme, in which the class probability and trajectory distribution were the most reasonable. The results of the study showed that MSM in the “high adherence decline group” had a higher overall adherence level (mean adherence >80%) and accounted for the largest proportion (64.13%), which indicated that this PrEP strategy had a high acceptability among MSM in Western China and that most MSM could successfully adhere to PrEP. Several previous studies have also explored patterns of change in adherence. For example, in the CROPrEP project conducted in China (Beijing, Shenyang, Shenzhen, and Chongqing), the researchers identified 2 types of adherence trajectories based on a group-based trajectory model, which were named “High adherence” and “Low adherence” [25]. In a randomized controlled study based on an MSM population in the United States, the authors identified subgroups of individuals with similar adherence trajectories, also based on growth mixture modeling [8]. At the same time, the trajectory for this initial state at a high level and subsequent decline in adherence have been seen in previous studies [10,11]. Previous studies have conducted qualitative interviews with this subset of the population and found that the most consistent topic of declining adherence was about the frequency of visits [11]. Users felt unsupported and overwhelmed during the long visit intervals, thus failing to adhere to their daily medication. These findings were consistent with evidence from previous studies, which suggested that visit frequency may be associated with medication adherence [26,27]. It is suggested that appropriately increasing the frequency of visits (follow-up) and giving encouragement and support to users will help to improve adherence and avoid a decline. In summary, our study used a person-centered approach based on the GMM that took into account individual differences and presented trajectories based on individual characteristics. Identifying these subgroups of discrete individuals who follow similar adherence trajectories can help identify those at risk for nonadherence who may require increased intervention support. We extended the literature on PrEP adherence by assessing patterns of change in PrEP adherence.

### Decision Tree to Predict Patterns of Change in Adherence

Over the past few years, machine learning methods have been increasingly used in a variety of fields and are equally feasible in small samples [28,29]. The MSM population is a sexual minority group and attaches greater importance to privacy issues, and we were unable to conduct a large-scale survey to get a large sample of data. Therefore, decision tree-based machine learning prediction method was more suitable for our study population. In addition, we combined the “intermediate adherence group” and “low adherence ascending group” identified by the GMM for prediction and prioritized the classification accuracy of this group. This decision was made to avoid incorrect negative categorization, which could result in MSM individuals with low adherence not receiving targeted interventions and guidance, increasing the risk of HIV infection. Indeed, many researchers advocated prioritizing sensitivity (classification accuracy for low and intermediate adherence groups) over specificity to ensure that the largest possible proportion of at-risk individuals were classified [30]. However, it should be noted that in our study, the classification accuracy of the “high adherence decline group” was 71.43%, which is acceptable in terms of specificity in addition to good sensitivity. Our study constructed a predictive model for PrEP adherence among the MSM population based on the decision tree and achieved good predictive results. We identified short questions and prediction rules that will facilitate the identification of individuals at risk for low adherence among PrEP users and targeted interventions that will be beneficial in improving adherence levels in high-risk populations.

### Factors Influencing Patterns of Change in Adherence Among MSM

At the same time, decision tree also provided insight and understanding of the predictor variables of the data. The variable in the root node was the most influential variable in the categorization of observations, while the other nodes contained variables that have influence on a subset of the data [31]. In the final model of the decision tree, we found that the HIV knowledge score (root node) was the most influential predictor variable, followed by educational attainment, group (reminder and nonreminder groups), and HIV testing. The results showed a significant correlation between HIV knowledge and adherence among the MSM population. According to the Information-Motivation-Behavioral Skills Model, knowledge had a significant impact on individual behavior change and guided HIV risk reduction interventions [32], which provided a theoretical explanation for our findings.

Our findings suggested that the mHealth intervention would be beneficial in improving PrEP medication adherence in the MSM population, which was also consistent with previous studies [33]. A daily mobile phone–based reminder is a promising intervention based on the actual needs and wishes of MSM. The importance of serving the unique needs and preferences of this marginalized population based on mHealth technology was also highlighted in a study in Nepal MSM [16]. In future studies, more multifaceted features and more reliable data collection approaches should be considered for mobile phone–based interventions. mHealth interventions should prioritize privacy and confidentiality, be intuitive, be user-friendly, and be tailored to the specific needs and preferences of MSM, thereby optimizing the functionality and acceptability of these mHealth care interventions.

Furthermore, Mujugira et al [34] mentioned in their study that HIV self-testing and PrEP are supplementary tools that can empower individuals to take control of their HIV protection. Regular HIV testing was recommended as part of the PrEP implementation guidelines. Our findings also suggested that HIV testing could provide an opportunity to promote PrEP adherence in the Chinese MSM population. Based on the results of the decision tree model, interventions targeting knowledge, medication reminders, and HIV testing would be beneficial in improving adherence in the MSM population.

### Limitations

Our study was a preliminary application of decision tree prediction modeling to PrEP adherence among MSM population in Western China. However, there are some limitations in our study. First, we measured adherence through participants’ self-reports, which may have been influenced by their recall bias, social desirability bias, and thus overestimation of adherence. However, a significant correlation between self-reported adherence and medication levels was found in a study of the PrEP program in a clinical setting, suggesting that self-reporting can be used to predict PrEP adherence [35]. Second, participants in our mHealth trial were rarely blinded, as they may have interacted with other participants in the survey field, thereby learning about the intervention, which is a typical shortcoming in mHealth trials. Although the results of our additional analyses indicated that there were no statistically significant differences between the intervention and control groups (Table S1 in Multimedia Appendix 1), the unblinded categorization of the 2 groups could have led to a potential impact on the results of the study. Future mHealth-based studies should fully consider the issue of blinding of interventions so as to ensure the accuracy of the findings. Meanwhile, there were the significant differences in baseline characteristics between included and excluded participants (Table S3 in Multimedia Appendix 1). According to the results of our analysis, age, place of residence, and employment status were statistically different in the inclusion and exclusion groups. The same shortcomings have been mentioned in previous studies [36]. The sample selected in their study was predominantly white and highly educated, which may have led to high estimates of self-reported adherence. However, we and previous researchers likewise agreed that such a bias was acceptable because these significantly different baseline characteristics were not the most significant factor influencing adherence. Meanwhile, for the evaluation metrics of the prediction model, we only selected 3 standard metrics and did not further adopt more metrics for assessment. However, the 10-fold cross-validation results had demonstrated the stability of the prediction results, and for small-sample machine learning predictions, we believed that the current metrics were sufficiently persuasive, and previous studies had used such an evaluation [22]. Since the MSM population is sensitive and concealed, this made our investigation process very difficult. Therefore, we did not have enough time to conduct external validation in this study. In future studies, we will validate the model more fully if the opportunity is available. Finally, further future research should be applied to larger samples while inputting more different variables so as to explore the accuracy of the predictive model.

### Conclusions

Awareness and utilization of PrEP are increasing in China, and information to support improved PrEP adherence is becoming more important. Our study modeled the longitudinal trajectory of adherence among the MSM population in Western China and predicted patterns of change in adherence. Accurately identifying patterns of change in adherence based on the decision tree and its predictive pathways can help administrators increase their focus on individuals who need preventive interventions that are conducive to improving PrEP adherence. This study was an important first step in implementing predictors of adherence among the MSM population using PrEP. More specifically, we proposed a short set of questions and prediction rules that could hopefully be assessed in future large-scale validation studies to prospectively predict patterns of change in adherence in high-risk populations. Meanwhile, interventions targeting knowledge, mHealth reminders, and HIV testing will also be beneficial in improving adherence among the MSM population. Future studies should continue to investigate the dynamic trajectories of adherence, assess patterns of change more frequently, and develop more accurate predictive models to better understand PrEP adherence in high-risk populations in the current environmental context.

## Supplementary material

10.2196/58920 Multimedia Appendix 1 Supplementary materials regarding presentation of the reminder messages on WeChat, comparison of basic information between the reminder and no-reminder groups, descriptive analysis of adherence, and univariate analysis of inclusion and exclusion groups.

10.2196/58920 Checklist 1 CONSORT-eHEALTH checklist (V 1.6.1).

## Abbreviations

aBIC: sample size–adjusted Bayesian information criteria
AIC: Akaike information criteria
BIC: Bayesian information criteria
Cp: complexity parameter
GMM: growth mixture model
mHealth: mobile health
MSM: men who have sex with men
PrEP: preexposure prophylaxis
