# Supplementary material for: Exploring and Predicting HIV Preexposure Prophylaxis Adherence Patterns Among Men Who Have Sex With Men: Randomized Controlled Longitudinal Study of an mHealth Intervention in Western China
Source: JMIR Mhealth Uhealth. 2024 Dec 12;12:e58920. doi: 10.2196/58920 (PMC11657909; doi:10.2196/58920)
Supplement: Multimedia Appendix 1 [file mhealth-v12-e58920-s001.docx]

**Supplementary materials**

**1. The presentation of the reminder messages on WeChat App**

In order to protect the user's privacy, the content of the messages we send were "processed". For example, when users received "You need to learn", it meant that they were reminded to take medication (Supplementary Figure 1-A). When users received "Time for examination", it meant that they were reminded to take medication (Supplementary Figure 1-B).


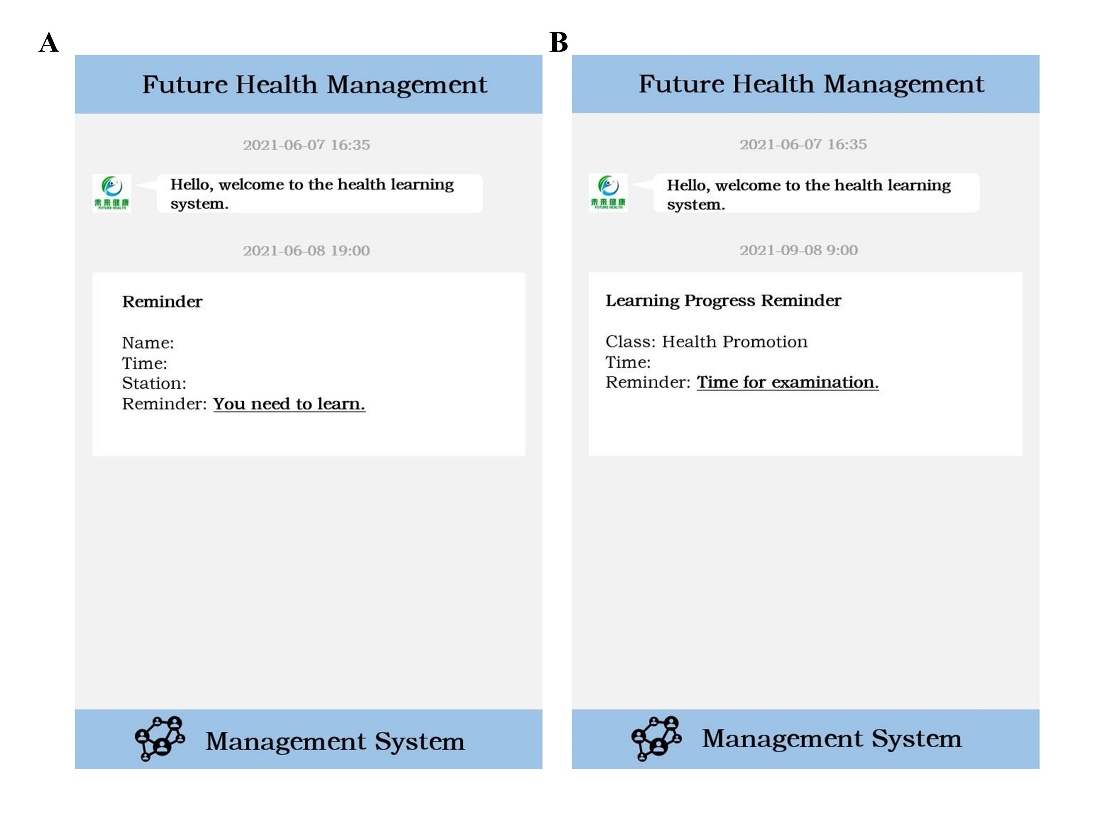


Supplementary Figure 1-A. The page of reminding users that they should take their medication.

Figure 1-B. The page of reminding users that they should participate in follow-up visits

The WeChat-based reminder system is also capable of recording adverse drug reactions (Supplementary Figure 2-A) and medication intake (Supplementary Figure 2-B). However, the participants in our study did not use this function and were recorded through the questionnaire. If the user has a serious adverse drug reaction, it is to seek help directly from the doctor and administrator.


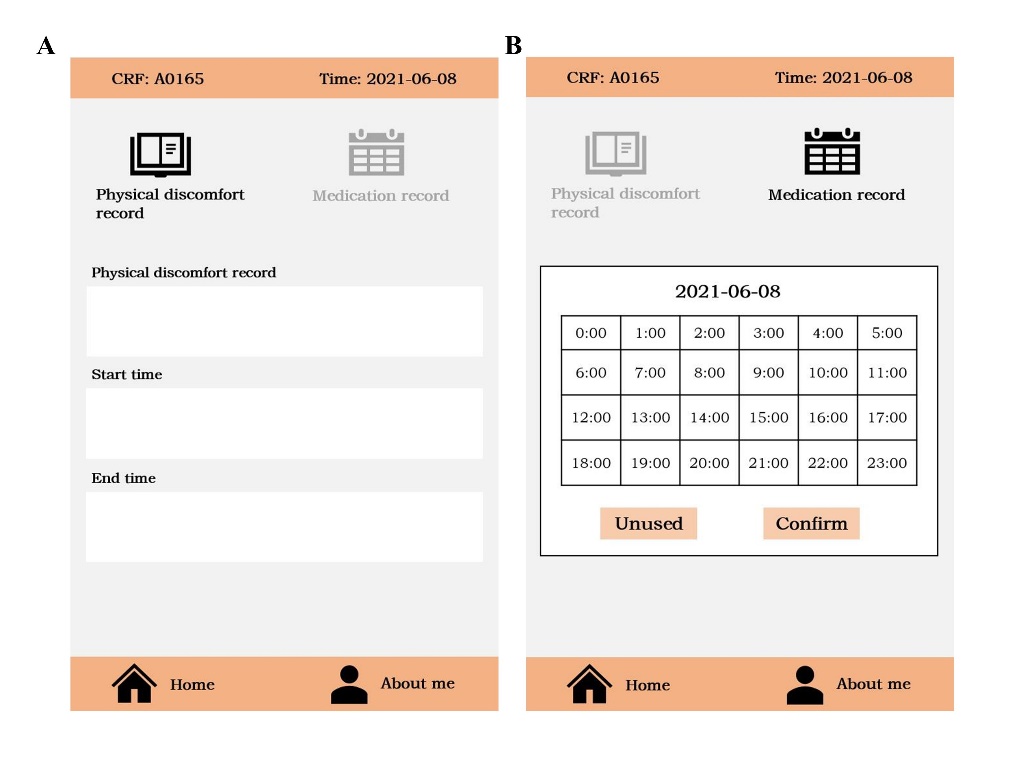


Supplementary Figure2-A. The page of recording adverse drug reactions of users.

Figure 2-B. The page of recording users taking medication

**2. Comparison of basic information between the reminder and no-reminder groups**

We compared basic demographic information between the reminder and no-reminder groups (Supplementary Table 1). According to the results of the univariate analysis, none of the variables differed statistically in both groups, suggesting that subgroups did not potentially influence the results.

Supplementary Table 1. Univariate analyses of the reminder and no-reminder groups

| Variables | Reminder group (N=234) | No-reminder group (N=212) | *P* value |
| --- | --- | --- | --- |
|  | N (%) | N (%) |  |
| Age | | | |
| 18-25 | 40 (17.09) | 30 (14.15) | .688 |
| 25-35 | 93 (39.74) | 86 (40.57) |  |
| ≥35 | 101 (43.17) | 96 (45.28) |  |
| Place of residence | | | |
| Urban | 167 (72.29) | 162 (76.78) | .281 |
| Rural | 64 (27.71) | 49 (23.22) |  |
| Ethnicity | | | |
| Ethnic Han | 220 (94.02) | 193 (91.04) | .230 |
| Ethnic minorities | 14 (5.98) | 19 (8.96) |  |
| Education attainment | | | |
| Primary school or below | 2 (0.85) | 2 (0.94) | .290 |
| Junior high | 8 (3.42) | 16 (7.55) |  |
| High school/Vocational high school | 50 (21.37) | 43 (20.28) |  |
| College/University degree or above | 174 (74.36) | 151 (71.23) |  |
| Employment status | | | |
| Employed | 204 (87.18) | 180 (84.90) | .786 |
| Unemployed/retired | 16 (6.84) | 17 (8.02) |  |
| Student | 14 (5.98) | 15 (7.08) |  |
| Marital status | | | |
| Married | 28 (11.97) | 32 (15.09) | .333 |
| Unmarried/divorced | 206 (88.03) | 180 (84.91) |  |
| Monthly income | | | |
| 1000-3000 CNY | 50 (21.46) | 54 (25.47) | .502 |
| 3000-10000 CNY | 168 (72.10) | 142 (66.98) |  |
| ≥10000 CNY | 15 (6.44) | 16 (7.55) |  |

CNY: Chinese Yuan.

**3. Descriptive analysis of adherence at each follow-up time point**

We performed descriptive analyses of the number of MSM and adherence in each follow-up period (Supplementary Table 2). The results of the study found that MSM decreased from an initial 446 to 137 during the follow-up period; the mean and standard deviation of their adherence changed from an initial 0.68±0.42 to 0.60±0.42; and the interquartile range of adherence changed from 0.18-1.00 to 0.14-1.00. The minimum lost to follow-up rate for the MSM population was 6.73% and the maximum was 35.13%, with an average lost to follow-up rate of 24.77%.

Supplementary Table 2. Descriptive analysis of adherence at each follow-up time point

| Follow-up time | N | Lost to follow-up rate  (%) | Adherence  Mean±SD | Adherence  IQR |
| --- | --- | --- | --- | --- |
| Time 1 | 446 | - | 0.68±0.42 | 0.18-1.00 |
| Time 2 | 416 | 6.73 | 0.72±0.39 | 0.50-1.00 |
| Time 3 | 316 | 24.04 | 0.69±0.40 | 0.29-1.00 |
| Time 4 | 205 | 35.13 | 0.65±0.42 | 0.14-1.00 |
| Time 5 | 137 | 33.17 | 0.60±0.42 | 0.14-1.00 |

SD: standard deviation; IQR: Interquartile range.

According to the results of the analysis, due to the long period of follow-up, the rate of loss of MSM individuals was high, decreasing from the initial 446 to 137, with an average loss rate of 24.77%. The results of the study were basically consistent with the lost to follow-up rate (29.5%) of the cohort of men who have sex with men in Harbin, China. In addition, the lost to follow-up rate of this investigation may be higher than that of related foreign studies. This may be due to the influence of traditional Chinese culture, where the MSM population is reluctant to disclose their identity for fear of discrimination, and thus cannot consistently participate in longitudinal investigations. At the same time, previous studies have also shown that the reasons for loss to follow-up in high-risk populations may be related to factors such as age and educational level *[Guevara Amanda, Coelho Lara, Veloso Valdiléa, et al. Travestis, transgender women and young MSM are at high risk for PrEP early loss to follow-up in Rio de Janeiro, Brazil. The Brazilian journal of infectious diseases: an official publication of the Brazilian Society of Infectious Diseases. 2022;27(1):102733]*. This suggests that in the process of future longitudinal studies in MSM population, emphasis should be given to the follow-up rate and the possible potential impact on the outcome variables.

**4. Univariate analysis of inclusion and exclusion groups**

We compared basic demographic information between the inclusion and exclusion groups (Supplementary Table 3). According to the results of our analysis, age, place of residence, and employment status were statistically different in the inclusion and exclusion groups.

Supplementary Table 3. Comparison of basic demographic characteristics of the inclusion and exclusion groups

| Variables | Exclusion group (N=176) | Inclusion group (N=446) | *P* value |
| --- | --- | --- | --- |
|  | N (%) | N (%) |  |
| Age | | | |
| 18-25 | 49 (27.84) | 70 (15.70) | <.001 |
| 25-35 | 72 (40.91) | 179 (40.13) |  |
| ≥35 | 55 (31.25) | 197 (44.17) |  |
| Place of residence | | | |
| Urban | 110 (63.58) | 329 (74.43) | .007 |
| Rural | 63 (36.42) | 113 (25.57) |  |
| Ethnicity | | | |
| Ethnic Han | 159 (90.34) | 413 (32.00) | .350 |
| Ethnic minorities | 17 (9.66) | 33 (7.40) |  |
| Education attainment | | | |
| Primary school or below | 2 (1.14) | 4 (0.90) | .569 |
| Junior high | 14 (7.95) | 24 (5.38) |  |
| High school/Vocational high school | 39 (22.16) | 93 (20.85) |  |
| College/University degree or above | 121 (68.75) | 325 (72.87) |  |
| Employment status | | | |
| Employed | 133 (75.57) | 384 (86.10) | <.001 |
| Unemployed/retired | 15 (8.52) | 33 (7.40) |  |
| Student | 28 (15.91) | 29 (6.50) |  |
| Marital status | | | |
| Married | 28 (16.00) | 60 (13.45) | .412 |
| Unmarried/divorced | 147 (84.00) | 386 (86.55) |  |
| Monthly personal income | | | |
| 1000-3000 CNY | 44 (25.14) | 104 (23.37) | .210 |
| 3000-10000 CNY | 112 (64.00) | 310 (69.66) |  |
| ≥10000 CNY | 19 (10.86) | 31 (6.97) |  |
| Group | | | |
| Reminder group | 85 (48.30) | 234 (52.47) | .348 |
| No-reminder group | 91 (51.70) | 212 (47.53) |  |

CNY: Chinese Yuan.
